# Dyadic Predictors of Willing to Engage in Physical Activity and Emotional Eating in Children and Adolescents with Mild and Moderate Intellectual Disability

**DOI:** 10.3390/nu15102343

**Published:** 2023-05-17

**Authors:** Kamila Czepczor-Bernat, Justyna Modrzejewska, Anna Porczyńska-Ciszewska, Adriana Modrzejewska, Izabela Bieńkowska, Paweł Matusik

**Affiliations:** 1 Department of Pediatrics, Pediatric Obesity and Metabolic Bone Diseases, Faculty of Medical Sciences in Katowice, Medical University of Silesia, 40-055 Katowice, Poland; endocrin@wp.pl; 2 Institute of Pedagogy, University of Bielsko-Biała, 43-309 Bielsko-Biala, Poland; jmodrzejewska@ath.bielsko.pl (J.M.); izabela.bienkowska@wp.pl (I.B.); 3 Faculty of Arts and Educational Sciences, University of Silesia, 40-007 Katowice, Poland; anna.porczynska@us.edu.pl; 4 Department of Psychology, Chair of Social Sciences and Humanities, School of Health Sciences in Katowice, Medical University of Silesia, 40-055 Katowice, Poland; adriana.modrzejewska@sum.edu.pl

**Keywords:** physical activity, emotional eating, body dissatisfaction, coping with emotions, feeding style, happiness, children, adolescents, parents, dyadic research, health education, health pedagogy

## Abstract

Intellectual disability is associated with increased risk for childhood obesity, and the factors most often associated with this risk are incorrect eating behavior and insufficient amount and intensity of physical activity. As is well known, there area whole range of factors determining lifestyle, but many currently available reports in this field refer to the functioning of children without a diagnosis of intellectual disability, and, as we know, due to numerous individual and environmental barriers, children with ID may function differently in this context than their peers. Therefore, we examined the relationships between the selected variables and divided them into two models: (1) first regression model: child’s willingness to engage in physical activity (dependent variable), child’s physical limitations related to disabilities and/or comorbidities, child’s independence, parents’ willingness to engage in physical activity, child’s body dissatisfaction (independent variables/predictors); (2) second regression model: child’s emotional eating (dependent variable), child’s coping with emotions, parents’ attitudes, beliefs, and practices about child feeding (restriction and pressure to eat), parents’ emotional eating, parents’ happiness (independent variables/predictors). A group of 503 parents (of children and adolescents with mild and moderate intellectual disability) completed: the Contour Drawing Rating Scale, the Child Feeding Questionnaire, the Emotional Overeating Questionnaire, the Scale of Experiencing Happiness, and the supplementary survey. Our results allow partial confirmation of the hypotheses related to both of these models: (1) model I: the relationships between the child’s willingness to engage in physical activity and all predictors are significant, but the direction of the relationship between the dependent variable and one of the predictors—body dissatisfaction—is opposite to the assumed one (negative relationship); (2) model II: the relationships between the child’s emotional eating and almost all predictors are significant, except for the relationship between the dependent variable and pressure to eat. In conclusion, (to the authors’ knowledge) this study is the first to assess dyadic predictors of willingness to engage in physical activity and emotional eating in children and adolescents with mild and moderate intellectual disability. It allows for a better understanding of the attitudes, beliefs, and experiences of children with intellectual disabilities and their parents, which gives the opportunity (taking into account factors from both individuals from the child-parent dyad) to better design strategies to support pro-health behaviors in children and adolescents from this group (which may improve the effectiveness of overweight prevention and obesity). These findings emphasize how important it is to consider the dynamic of the child-parent dyad when considering how parenting contributes to a child’s willingness to engage in physical activity, as well as thatchild’s emotional eating.

## 1. Introduction

The definition of intellectual disability (ID) given by the American Association on Intellectual and Developmental Disabilities (AAIDD) indicates a condition characterized by significant limitations in intellectual functioning, as well as in adaptive behavior, which includes many social and practical skills developed before the age of 22 [1]. In this context, there are also reports in the literature that intellectual disability is associated with increased risk for childhood obesity, and the factors most often associated with this risk are incorrect eating behavior and insufficient amount and intensity of physical activity [2]. As is well known, there area whole range of factors determining lifestyle, and these will be both individual factors (e.g., beliefs about oneself and one’s body; physical limitations related to disabilities and/or comorbidities) and environmental (e.g., including family ones—parental beliefs, attitudes, and practices regarding child feeding, motivating the child to a healthy lifestyle, and maintaining such a healthy lifestyle by the parents) [3,4]. However, what is important in this context is that many currently available reports in this field refer to the functioning of children without a diagnosis of intellectual disability, and, as we know, due to numerous individual and environmental barriers, children with ID may function differently in this context than their peers [5,6]. Therefore, using the perspective of dyadic research (based on data from both individuals of the child-parent pair), it is worth analyzing in more detail from which selected variables of the child’s and parents’ functional range we can predict children’s willingness to engage in physical activity and the tendency to eat under the influence of emotions. Such research seems to be important because: (1) to the authors’ knowledge, there is a lack of more detailed dyadic analyses of the willingness to engage in physical activity and emotional eating in children with intellectual disabilities, (2) dyadic research may enable us to gain a deeper and fuller (richer) understanding of attitudes, beliefs, and experiences of study participants than a single perspective, and (3) these findings may allow to create more effective overweight and obesity prevention strategies among children with ID.

It is well known that physical activity has a beneficial effect on people with intellectual disabilities [7]. Physical activity improves both static and dynamic balance [8] and overall skill-based fitness [9]. The benefits of physical activity are crucial for children with intellectual disabilities, due to the presence of a variety of problems related to physical health, motor skills, and the presence of psychological issues [10]. The World Health Organization (WHO) [11] recommends that children and young people with disabilities undertake moderate to vigorous physical activity for at least 60 min a day throughout the week, with three intensive days. Maintaining such recommendations also seems to be crucial, due to the fact that previous research has shown that physical activity has a positive effect on psychosocial well-being and emotional problems in children and adolescents (i.e., [7,10,12]). As mentioned above, many factors may determine the willingness to engage in physical activity. The obvious factors we should consider when assessing the willingness to engage in physical activity in children with ID seem to be the child’s physical limitations related to disabilities and/or comorbidities and their independence [6,13,14]. However, in addition to these factors, it should also be mentioned that an important factor influencing the participation of children with intellectual disabilities in physical activity is their parents [15,16]. Previous research has shown that the behavior of parents of children with intellectual disabilities related to physical activity, and their beliefs on this subject, are closely related to their children’s activity [17]. This is due, among other things, to the fact that (according to social learning theory, proposed by Albert Bandura, [18,19]) children learn not only through rewards and punishments (for performing certain activities), but also (to a large extent) through observation and imitation. Therefore, the willingness to engage in physical activity presented by the parents themselves can be very important for shaping this willingness in children. At the end of the discussion of selected predictors of the willingness to engage in physical activity, perhaps it is worth mentioning one more important variable—body dissatisfaction. It seems that it would be interesting to include this variable in the analysis, because, so far, many studies have been conducted among children and adolescents without ID on the relationship between body dissatisfaction and physical activity (some studies indicate a positive relationship [20], and others negative [21]), but (to the authors’ knowledge) there are still few analogous studies in children and adolescents with ID.

Equally important as physical activity for children with intellectual disabilities is their adequate intake of energy and nutrients, which play a very important role in their development [22]. Balanced (healthy) eating behavior involves, among other things, starting and ending a meal in response to hunger and satiety signals [23,24]. However, in many studies, (over)eating in the absence of hunger is increasingly being observed in children’s eating patterns, which may be related to the emotional state of the child and the lack or insufficient flexibility in dealing with emotions effectively [25]. In this context, research proves that psychological stress changes attitudes to eating towards unhealthy choices, and its consequence can be emotional eating (i.e., [26,27]). Considering the research group of children with intellectual disabilities, most of the research concerns feeding problems [28], nutritional status [29], being overweight, and obesity [30]. The study of emotional eating in children is difficult because parents: (1) still largely control their children’s food choices [31], (2) (according to a controlling/authoritarian feeding style) may be convinced that children do not fully know what and how much they should eat, therefore significant (often rigid) restrictions should be introduced and pressure should be put on them to finish their meals [32], and (3) often use rewards in the form of unhealthy snacks [33]. Such practices result in children reaching for unhealthy snacks more often in response to negative emotions [23], and, overall, children are at an increased risk of overeating and undereating (e.g., in order to comply with their parents’ requests—regardless of the signals of hunger and satiety—children eat everything on their plates; children feel strong stress and aversion to products, due to pressure from their parents), which can cause serious weight problems (both underweight and overweight/obesity) [24,32]. When analyzing the predictors of emotional eating in children, two more issues are worth mentioning. Firstly, research confirms that parents’ emotional eating is also positively related to their children’s emotional eating [34] and parents who are characterized by emotional eating also apply more emotion-regulating eating practices to their children [35]. Secondly, it may be interesting to verify whether a parent’s emotional state (and, in particular, happiness) can be a predictor of emotional eating in a child (it can be concluded that a parent’s emotional state on a daily basis will affect their child’s emotional functioning and will affecthow the child’s process of identifying and managing emotions will proceed; this is known in many children with intellectual disabilities. The processes described above, related to emotional functioning, may create the emergence of some additional difficulties and barriers [36,37]).

To sum up, in the present study, we examined the predictors of children’s willingness to engage in physical activity and emotional eating in children and adolescents with mild and moderate intellectual disability. Variables related to the functioning of the children themselves (physical limitations related to disabilities and/or comorbidities, independence, body dissatisfaction, coping with emotions) and their parents (willingness to engage in physical activity, feeding-related practice, emotional eating, happiness) were taken into account as predictors. Therefore, in the main analysis, we examined the relationships between all the above-mentioned variables and divided them into two models: (1) model I: child’s willingness to engage in physical activity (dependent variable), child’s physical limitations related to disabilities and/or comorbidities, child’s independence, parents’ willingness to engage in physical activity, child’s body dissatisfaction (independent variables/predictors); (2) model II: child’s emotional eating (dependent variable), child’s coping with emotions, parents’ attitudes, beliefs, and practices about child feeding (restriction and pressure to eat), parents’ emotional eating, parents’ happiness (independent variables/predictors). In our main hypotheses, we assumed that: (1) the first hypothesis—model I: the higher levels of the last two of the above-mentioned variables (parents’ willingness to engage in physical activity and child’s body dissatisfaction) would be associated with higher willingness of the child to engage in physical activity, after accounting for the effects of foregoing the child functioning-related variables (physical limitations related to disabilities and/or comorbidities and child’s independence) and; (2) the second hypothesis—model II: the higher levels of the parental feeding-related practice and parents’ emotional eating and the lower level of the parents’ happiness would be associated with the child’s emotional eating, after accounting for the effect of foregoing the child functioning-related variable (child’s coping with emotions).

## 2. Materials and Methods

### 2.1. Participants and Procedure

Our cross-sectional study complies with the Declaration of Helsinki and was performed according to Ethics Committee approval (no. KEUS 239/04.2022). Participants were recruited by disseminating information about the possibility of participating in the study in places where medical practitioners, psychologists, pedagogists, and other healthcare providers can meet the parent of a child with an intellectual disability (e.g., support groups, centers for the treatment of diseases associated with disability, psychological and pedagogical counselling centers etc.) in 2022. A non-probability sampling technique was used (a voluntary method of sampling). Each parent interested in participating in the study was initially informed about its purpose and that the study was voluntary and anonymous. Participants who were ultimately interested in participating in the study, after contacting the investigator, received informed consent to participate in the study for approval. They then completed the measures described in the next section of this manuscript. Parents were not remunerated for their participation. At this point, it should be remembered that it was the parents who completed the answers regarding the functioning of themselves and their children in all the measures used. Initially, 675 parents signed up for the study (inclusion criterion in the study: children and adolescents with intellectual disability). Finally, a group of 503 parents of children and adolescents with mild and moderate intellectual disability was selected for analysis in this manuscript (exclusion criterion from the analysis presented in this article: children and adolescents with severe and profound intellectual disabilities were excluded from the analysis, due to the fact that these levels significantly may limit contact with the child and/or establishing cooperation, e.g., in the context of lifestyle-related behaviors, and, very often, these levels of intellectual disability may be accompanied by significant issues that make it difficult to move and/or engage in physical activity). Table 1 presents the detailed characteristics of the group.

### 2.2. Measures

#### 2.2.1. The Supplementary Survey

This measure included:

(1) child’s willingness to engage in physical activity—to what extent is the child willing to engage in physical activity (on a scale from 0—unwillingly to 100—most willingly)?
(2) child’s physical limitations related to disabilities and/or comorbidities—how strong are the child’s physical limitations resulting from disabilities and/or comorbidities (on a scale from 0—no physical limitations to 100—very strong physical limitations)?
(3) child’s independence—child’s independence (on a scale from 0—completely dependent to 100—completely independent);
(4) parents’ willingness to engage in physical activity—how willing are you to engage in physical activity (on a scale from 0—unwilling to 100—most willing)?
(5) child’s emotional eating—does the child overeat under the influence of experiencing negative emotions (dichotomous variable: 1—yes, 0—no);
(6) child’s coping with emotions—on a scale of 0 (completely bad) to 100 (completely good), rate how your child copes with emotions in everyday life;
(7) sociodemographic and other variables—gender, education, levels of an intellectual disability, financial status, professional activity, age, weight, height.

#### 2.2.2. Child’s Body Dissatisfaction: The Contour Drawing Rating Scale (CDRS) [38]

This scalecontains 9 silhouettes (ranking from the thinnest to the fattest) [38]. Parents were asked to answer two questions: (1) Which silhouette reflects what your child looks like now? (real body image); (2) Which silhouette represents what your child would like to look like? (ideal body image). The higher the discrepancy between ideal and real body image, the greater the body dissatisfaction. Previous studies indicate adequate test-retest reliability, e.g., [38].

#### 2.2.3. Restriction and Pressure to Eat: The Child Feeding Questionnaire (CFQ) [39]

This 31-item questionnaire includes seven subscales: restriction, pressure to eat, monitoring, perceived responsibility, parent perceived weight, perceived child weight, parents’ concerns about child weight [39]. This study focused on two of them—restriction (e.g., I intentionally keep some foods out of my child’s reach) and pressure to eat (e.g., My child should always eat all of the food on her plate). Participants answer on a 5-point scale (changing depending on the subscale/question). The higher scores reflect stronger parental attitudes, beliefs, and practices about child feeding. A Polish version of the instrument was previously translated (a forward-backward method). Previous studies indicate adequate reliability and validity, e.g., [39]. Cronbach’s alpha coefficient (internal consistency) was: (1) restriction = 0.85, (2) pressure to eat = 0.78.

#### 2.2.4. Parents’ Emotional Eating: The Emotional Overeating Questionnaire (EOQ) [40]

This 9-item questionnaire shows how often people have eaten excessive amounts of food over the last 28 days when they feel: sadness, loneliness, anxiety, anger, tiredness, happiness, guilt, boredom, and physical pain [40]. The scores range from 0 (no days) to 6 (everyday). The higher the score, the higher the emotional overeating. A Polish version of the instrument was previously translated (a forward-backward method). Previous studies indicate adequate reliability and validity, e.g., [40]. Cronbach’s alpha coefficient (internal consistency) was 0.90.

#### 2.2.5. Parent’s Happiness: The Scale of Experiencing Happiness/Skala Doświadczania Szczęścia (SDS) [41]

This Polish 40-item questionnaire reflects the level of happiness (e.g., I feel life is worth living) [41]. The scores range from 1 (never) to 7 (always). The higher the score, the higher the happiness. Previous studies indicate adequate reliability and validity, e.g., [41]. Cronbach’s alpha coefficient (internal consistency) was 0.97.

### 2.3. Statistical Analysis

The relationships between all variables were analyzed by Pearson’s correlation coefficient. This analysis was a preparatory step for regression analyses. Hierarchical regressions were used to verify our hypotheses.

In the first regression model (model I), the child’s willingness to engage in physical activity was a criterion variable. As a first step, we entered the child’s physical limitations related to disabilities and/or comorbidities and the child’s independence. As a second step, we entered the parents’ willingness to engage in physical activity, and, as a third step, we entered the child’s body dissatisfaction. In this way, it was possible to examine the extent to which parental willingness to engage in physical activity and child body dissatisfaction incrementally predicted the child’s willingness to engage in physical activity, after accounting for variance associated with the other variables (child’s physical limitations related to disabilities and/or comorbidities and their independence). In the second regression model (model II), the child’s emotional eating was a criterion variable. As a first step, we entered the child’s ability to cope with emotions. As a second step, we entered two variables related to parents’ attitudes, beliefs, and practices about child feeding (restriction and pressure to eat), and, as a third step, we entered parents’ emotional eating and parents’ happiness. In this way, it was possible to examine the extent to which parental feeding-related practice, and parents’ emotional eating and happiness incrementally predicted the child’s emotional eating, after accounting for variance associated with the child’s coping with emotions.

For both models, the main assumptions underlying multiple regression models have been met. Variance inflation factors did not exceed 10 and tolerance was higher than 0.2 [42,43].

## 3. Results

### 3.1. Preliminary Analyses

Pearson correlation coefficient and descriptive statistics (M and SD) for all analyzed variables in the first and second regression models are presented in Table 2 and Table 3.

### 3.2. Regression Analyses

The regression analyses results are shown in Table 4 and Table 5. Summarizing the results: (1) model I: it turned out that all predictors are significantly associated with the dependent variable (child’s willingness to engage in physical activity), but the relationship between the dependent variable and the child’s body dissatisfaction is in the opposite direction to the assumed one; (2)model II: it was found that almost all predictors (except pressure to eat) are significant associated with the dependent variable (child’s emotional eating) and directions of all relationships between the dependent variable and our predictors are as expected.

## 4. Discussion

The main objective of our study was to assess the relationships in the two following regression models related to the functioning of children and adolescents with mild and moderate intellectual disability (ID): (1) model I: child’s willingness to engage in physical activity (dependent variable), child’s physical limitations related to disabilities and/or comorbidities, child’s independence, parents’ willingness to engage in physical activity, child’s body dissatisfaction (independent variables/predictors); (2) model II: child’s emotional eating (dependent variable), child’s coping with emotions, parents’ attitudes, beliefs, and practices about child feeding (restriction and pressure to eat), parents’ emotional eating, parents’ happiness (independent variables/predictors). Our results allow partial confirmation of the hypotheses related to both of these models: (1) model I: the relationships between the child’s willingness to engage in physical activity and all predictors are significant, but the direction of the relationship between the dependent variable and one of the predictors—body dissatisfaction—is opposite to the assumed one (negative relationship); (2) model II: the relationships between the child’s emotional eating and almost all predictors are significant, except for the relationship between the dependent variable and pressure to eat.

With regard to the relationships in the first regression model, it turns out that the increasing level of the child’s willingness to engage in physical activity can be predicted on the basis of a decrease in the level of the child’s physical limitations (related to disabilities and/or comorbidities) and body dissatisfaction, and an increase in the level of the child’s independence and parents’ willingness to engage in physical activity. Our findings seem to be consistent with previous reports, which show that such basic factors as the child’s physical limitations (related to disabilities and/or comorbidities) or child’s independence largely determine various behaviors of children and adolescents with ID (including those related to physical activity) [6,13,14]. This study also confirmed the role of parental modelling in a child’s willingness to engage in physical activity in the group with ID [44]. Interestingly, however, it turned out that the relationship between the child’s willingness to engage in physical activity and dissatisfaction is negative, which is consistent with the results (related to adolescence) obtained by Gualdi-Russo et al. [21], and the opposite of the results obtained by Miranda et al. [20]. At this point, it should be noted that, although it was assumed that greater body dissatisfaction would be associated with a growing child’s willingness to engage in physical activity (as a method of changing body shape and/or weight), it turns out that perhaps a lower level of body dissatisfaction is associated with a greater level of a child’s willingness to engage in physical activity, because children with a more positive body image and a higher appreciation of their bodies are more likely to engage in physical activity and enjoy it more. However, further research should be conducted in this context, which will take into account important mediators/moderators (e.g., gender, body weight, perceived social pressure to change body weight/figure) and analyze other aspects of body perception in more detail (e.g., body functionality, positive body image [21,45]).

Considering the relationships in the second regression model, it turns out that an increasing level of a child’s emotional eating can be predicted on the basis of a decrease in the level of a child’s coping with emotions, as well as their parents’ happiness, and an increase in the level of restriction and parents’ emotional eating (but not based on pressure to eat, which turned out to be an insignificant predictor of a child’s emotional eating). Our results are consistent with previous reports that indicated a similar positive relationship between emotional eating in children without ID and their parents (e.g., [46]); the basic mechanism described as underlying this relationship is parental modelling [18]. Although our research is not longitudinal, and conclusions should be drawn carefully from it, it should be emphasized that this observation is important because it may indicate a parental contribution to the tendency to overeat in response to various emotions, regardless of the level of hunger and satiety [47]. With regard to the relationship between a child’s emotional eating and their coping with emotions, emotional eating may become a permanent or (very often) chosen strategy of coping with emotions, which, given the difficulties in coping effectively with other methods, may be a behavior that increases the risk of weight gain [48]. These increased risks are also related to the fact that emotional eaters often tend to choose high-energy foods (perceived as tasty), which can effectively stimulate hedonistic pleasure and thus distract from unpleasant stimuli/situations [49,50]. Perhaps, therefore, it could mean that, due to the greater risk of difficulties in emotional functioning in children with intellectual disabilities (e.g., due to lower levels of impulse control and effortful control), emotional eating may be a strategy for dealing with emotions that are difficult for the child when they have the opportunity to observe a lowered mood and lowered level of happiness on a daily basis in their parents (which also seems to be consistent with our finding about the relationship between a child’s emotional eating and their parents’ happiness in previous findings) [36,37]. When analyzing the last of the significant relationships between a child’s emotional eating and restrictions, it should be mentioned that similar relationships have already been described in earlier studies among children without ID, which indicated that a controlling/authoritarian feeding style can be a significant source of stress for the child (e.g., due to numerous restrictions and the need to constantly control eating) and teach the child (unintentionally) to eat without taking into account hunger and satiety signals [23,39,51]. Interestingly, however, it was not possible to confirm a significant relationship between the pressure to eat and emotional eating, and further research is needed to more thoroughly analyze the mechanism underlying the relationship between these variables. Perhaps there are significant moderators/mediators of this relationship (e.g., intensity of negative affect, child’s body weight; [24]).

Referring to the findings described above, some researchers have analyzed physical activity, eating patterns, and body mass index among children and adolescents with ID [52,53,54,55], indicating the importance of adequately intense activity and healthy eating habits in limiting weight gain in this group. This is important because the above studies, as well as cross-sectional studies conducted among Chinese, American, and English children, have shown that children with intellectual disabilities are more likely to be overweight and obese, have nutritional problems, and are significantly less active compared to children without disabilities [30,52,56,57]. Undoubtedly, both physical activity and eating behaviors may affect the emotional state (which may significantly affect the level of motivation related to maintaining a healthy lifestyle [58]). Therefore, further research should also analyze these relationships and analyze the motives for undertaking physical activity and eating behaviors in both individuals of the child-parent dyad. This is important because it is mental well-being and a sense of happiness that enables a parent to be fully involved in raising their child and gives them the strength to provide loving, accepting, patient, and kind care for their child. Undoubtedly, a parent’s positive emotions are a resource that generates their children’s experience of positive emotions, and, as Seligmann [59] points out, the consequence of positive emotions is to increase and develop the child’s intellectual, physical, and social resources, which, in turn, provides the foundation for the psychological well-being of every young person, including those with intellectual disabilities.

Our study had several important limitations, mainly: (1) it was a cross-sectional design; (2) it used only self-report measures (based on the parents’ assessment of their children’s functioning); (3) although our sample consisted of fathers and mothers, there was a significant advantage of mothers; (4) a convenience sampling method was used to recruit participants; (5) (despite good reliability and validity of all measures) not all questionnaires have been previously validated in a Polish sample; (6) our study did not focus on a detailed assessment of a child’s IQ (with a distinction between different areas where a child may have slightly different levels of skill and development; parents, on the basis of a previous diagnosis, informed about the level of intellectual disability of the child); (7) neurologic conditions in comorbidity or the intellectual disability due to syndromes were not excluded or considered in detail (which, as you know, may be important, because these aspects can affect the problem of weight or eating; for example, in some genetic syndromes—such as Prader-Willi Syndrome or Bardet-Biedl Syndrome—the feeding management is problematic for parents, due to the typical voracity of these children); (8) caregivers’ burdenrelated to child’s intellectual disability was not analyzed, and, as is known, parents play a central role in the education and rehabilitation processes (therefore, when their child requires additional and/or long-term support, and/or when parents do not have support from other important people or institutions (i.e., special school/kindergarten), they may be at a much greater risk of developing psychosocial problems, including severe stress, depression, and parental burnout) [60,61,62].It is therefore necessary to conduct (longitudinal or experimental) research in the future that will take into account the above limitation, so that there are studies that indicate, among others, that the parental feeding style may be decisive for the level of the child’s emotional eating, and the relationship between these variables is bidirectional (e.g., [63]). Further research (with the introduction of improvements to the project) is also necessary, due to the fact that, by using objective measurements, we will avoid bias in response, and by using ecological momentary assessment (EMAs; measurement based on repeated measurement of variable(s) in an individual’s natural environment) we will gain even better insight into the exact dynamics of the relationship between variables and ongoing changes.

## 5. Conclusions

As mentioned above, our findings partially confirm our hypotheses: (1) the relations between a child’s willingness to engage in physical activity and all predictors are significant (only the relationship with body dissatisfaction is opposite to the assumed one); (2) the relationship between the child’s emotional eating and almost all predictors are significant (except for the relationship with pressure to eat). In conclusion, using the perspective of dyadic research (based on data from both persons from the child-parent pair), it was analyzed in more detail how we can predict (on the basis of selected variables of the child’s and parents’ functional range) children’s tendenciesto engage in physical activity, and the tendency in children and adolescents with mild and moderate intellectual disabilityto eat under the influence of emotions. Such studies seem important because (as mentioned above): (1) the vast majority of previously available reports in the analyzed field refer to the functioning of children without a diagnosis of intellectual disability (and, as we know, due to numerous individual and environmental barriers, children with intellectual disabilities may function differently in this context than their peers); (2) these dyadic studies allow for a deeper and fuller (richer) understanding of the attitudes, beliefs, and experiences of the study participants than a single perspective; (3) these results may be the basis for creating equitable healthcare quality and safety for children and adolescents with ID.

To the authors’ knowledge, this study is the first to assess dyadic predictors of willingness to engage in physical activity and emotional eating in children and adolescents with mild and moderate intellectual disability. Moreover, it allows for a better understanding of the attitudes, beliefs, and experiences of children with intellectual disabilities and their parents, which gives the opportunity (taking into account factors from both individuals from the child-parent dyad) to better design strategies to support pro-health behaviors in children and adolescents from this group (which may improve the effectiveness of overweight prevention and obesity). These findings emphasize how important it is to consider the dynamic of the child-parent dyad when considering how parenting contributes to a children’s willingness to engage in physical activity and children’s emotional eating. As the prevalence of children’s emotional eating is increasing (e.g., [64]) and statistics on physical activity show a decline among children and adolescents (e.g., [65]), it is crucial that interventions for children consider the role of parental functioning and their beliefs, attitudes, and practices regarding child feeding alongside the individual characteristics of children.

## Figures and Tables

**Table 1:** Socio-demographic characteristics of both individuals of the parent-child dyad.

	Children and Adolescents	Parents
	***N* (%)**	
Gender	Female: 248 (49.30%) Male: 255 (50.70%)	Female: 414 (82.31%) Male: 89 (17.69%)
Education		Elementary: 16 (3.18%) Vocational: 95 (18.89%) Secondary: 182 (36.18%) Higher: 210 (41.75%)
Levels of an intellectual disability	Mild: 359 (%) Moderate: 144 (%)	
Financial status		Below average: 34 (6.76%) Average: 372 (73.96%) Above average: 97 (19.28%)
Professional activity		Full-time job: 288 (57.26%) Part-time job: 92 (18.29%) Odd/casual job: 32 (6.36%) Lack of work: 91 (18.09%)
	***M* (*SD*)**	
Age	10.17 (3.80)	38.43 (7.03)
Weight (kg)	41.92 (18.24)	71.19 (15.52)
Height (cm)	139.81 (20.72)	168.97 (7.68)
BMI (kg/m^2^)	20.48 (5.25)	24.88 (4.99)

**Table 2:** The first regression model (model I): correlation, mean, and standard deviation.

	1	2	3	4	5
1. Child’s willingness to engage in physical activity		−0.42 ***	0.39 ***	0.31 ***	−0.29 ***
2. Child’s physical limitations related to disabilities and/or comorbidities			−0.44 ***	−0.11 *	0.18 **
3. Child’s independence				0.15 **	−0.09 *
4. Parents’ willingness to engage in physical activity					−0.10 *
5. Child’s body dissatisfaction					
***M* (*SD*)**	64.12 (31.69)	32.03 (29.40)	60.13 (27.48)	58.30 (32.61)	1.34 (1.25)

* *p* < 0.05, ** *p* < 0.01, *** *p* < 0.001.

**Table 3:** The second regression model (model II): correlation, mean, and standard deviation.

	1	2	3	4	5	6
1. Child’s emotional eating		−0.23 ***	0.31 ***	0.05	0.25 ***	−0.24 ***
2. Child’s coping with emotions			−0.18 ***	−0.10 *	−0.20 ***	0.13 **
3. Restriction				0.16 ***	0.18 ***	0.04
4. Pressure to eat					0.25 ***	−0.17 ***
5. Parents’ emotional eating						−0.41 ***
6. Parents’ happiness						
***M* (*SD*)**	None ^1^	53.56 (24.34)	3.18 (1.00)	2.61 (1.14)	0.88 (1.00)	179.39 (33.22)

* *p* < 0.05, ** *p* < 0.01, *** *p* < 0.001. ^1^ Dichotomous variable—*Does child overeat under the influence of experiencing negative emotions* (1—yes, 0—no).

**Table 4:** Results of hierarchical regression analysis for the prediction of child’s willingness to engage in physical activity (model I).

		Child’s Willingness to Engage in Physical Activity				
Step	Variables	B	SE	*β* [95% CI]	*t*	*p*
1		*F*(2, 502) = 73.61, *p* < 0.001, Adj. *R*^2^ = 0.22				
	Child’s physical limitations related to disabilities and/or comorbidities	−0.33	0.05	−0.31 [−0.41–−0.21]	−7.09	*p* < 0.001
	Child’s independence	0.29	0.05	0.25 [0.15–0.35]	5.79	*p* < 0.001
2		*F*(3, 502) = 66.16, *p* < 0.001, Adj. *R*^2^ = 0.29 (Δ*Fp* < 0.001)				
	Child’s physical limitations related to disabilities and/or comorbidities	−0.32	0.05	−0.30 [−0.40–−0.20]	−7.00	*p* < 0.001
	Child’s independence	0.26	0.05	0.22 [0.12–0.32]	5.27	*p* < 0.001
	Parents’ willingness to engage in physical activity	0.24	0.04	0.24 [0.16–0.32]	6.31	*p* < 0.001
3		*F*(4, 502) = 59.04, *p* < 0.001, Adj. *R*^2^ = 0.32 (Δ*Fp* < 0.001)				
	Child’s physical limitations related to disabilities and/or comorbidities	−0.28	0.05	−0.26 [−0.36–−0.16]	−6.31	*p* < 0.001
	Child’s independence	0.26	0.05	0.22 [0.12–0.32]	5.36	*p* < 0.001
	Parents’ willingness to engage in physical activity	0.22	0.04	0.23 [0.15–0.31]	6.06	*p* < 0.001
	Child’s body dissatisfaction	−4.99	0.96	−0.20 [−2.09–1.69]	−5.22	*p* < 0.001

**Table 5:** Results of hierarchical regression analysis for the prediction of child’s emotional eating (model II).

		Child’s Emotional Eating				
Step	Variables	B	SE	*β* [95% CI]	*t*	*p*
1		*F*(1, 502) = 26.62, *p* < 0.001, Adj. *R*^2^ = 0.05				
	Child’s coping with emotions	−0.004	0.001	−0.23 [−0.23–−0.23]	−5.16	*p* < 0.001
2		*F*(3, 502) = 23.82, *p* < 0.001, Adj. *R*^2^ = 0.12 (Δ*Fp* < 0.001)				
	Child’s coping with emotions	−0.003	0.001	−0.18 [−0.18–−0.17]	−4.13	*p* < 0.001
	Restriction	0.13	0.02	0.28 [0.24–0.32]	6.51	*p* < 0.001
	Pressure to eat	−0.007	0.02	−0.02 [−0.06–0.02]	−0.39	*p* > 0.05
3		*F*(5, 502) = 21.91, *p* < 0.001, Adj. *R*^2^ = 0.17 (Δ*Fp* < 0.001)				
	Child’s coping with emotions	−0.003	0.001	−0.14 [−0.14–−0.13]	−3.30	*p* < 0.01
	Restriction	0.12	0.02	0.27 [0.23–0.31]	6.34	*p* < 0.001
	Pressure to eat	−0.03	0.02	−0.07 [−0.11–−0.03]	−1.59	*p* > 0.05
	Parents’ emotional eating	0.05	0.02	0.11 [0.07–0.15]	2.43	*p* < 0.05
	Parents’ happiness	−0.002	0.001	−0.18 [−0.18–−0.17]	−3.94	*p* < 0.001

## Data Availability

The data that support the findings of this study are available from the corresponding author upon reasonable request.
